# USPs in Pancreatic Ductal Adenocarcinoma: A Comprehensive Bioinformatic Analysis of Expression, Prognostic Significance, and Immune Infiltration

**DOI:** 10.1155/2022/6109052

**Published:** 2022-12-20

**Authors:** Yizhi Wang, Dongkai Zhou, Yang Kong, Qifan Yang, Yuan Ding, Weilin Wang

**Affiliations:** ^1^Department of Hepatobiliary and Pancreatic Surgery, The Second Affiliated Hospital, School of Medicine, Zhejiang University, Hangzhou 310009, China; ^2^Key Laboratory, Precision Diagnosis and Treatment for Hepatobiliary and Pancreatic Tumor of Zhejiang Province, Hangzhou 310009, China; ^3^Research Center, Diagnosis and Treatment Technology for Hepatocellular Carcinoma of Zhejiang Province, Hangzhou 310009, China; ^4^Clinical Medicine Innovation Center, Precision Diagnosis and Treatment for Hepatobiliary and Pancreatic Disease, Zhejiang University, Hangzhou 310009, China; ^5^Clinical Research Center of Hepatobiliary and Pancreatic Diseases of Zhejiang Province, Hangzhou 310009, China; ^6^Cancer Center, Zhejiang University, Hangzhou 310009, China

## Abstract

Pancreatic ductal adenocarcinoma (PDAC), as an intractable malignancy, still causes an extremely high mortality worldwide. The ubiquitin-specific protease (USP) family constitutes the major part of deubiquitinating enzymes (DUBs) which has been reported to be involved in initiation and progression of various malignancies via the function of deubiquitination. However, the biological function and clinical values of USPs in PDAC have not been comprehensively elucidated. In this study, Gene Expression Profiling Interactive Analysis (GEPIA), Gene Expression Omnibus (GEO) datasets, UALCAN database, and the Human Protein Atlas (HPA) online tool were used to analyze the expression level and the relationship between USP expression and clinicopathological features in PDAC. Survival module of HPA and Kaplan-Meier plotter (KMP) databases was recruited to explore the prognostic value of USPs. Tumor Immune Estimation Resource (TIMER) online tool and KMP databases were utilized to elucidate tumor immune infiltration and immune-related survival of USPs. CBioPortal online tool was used to identify the gene mutation level of USPs in PDAC. Both cBioPortal and LinkedOmics were used to confirm the potential biological functions of USPs in PDAC. Our study showed that USP10, USP14, USP18, USP32, USP33, and USP39 (termed as six-USPs) expressions were significantly elevated in tumor tissues. The high expression of the four USPs (USP10, USP14, USP18, and USP39) indicated a poor prognosis. A significant relationship was indicated between the expression of six-USPs and clinicopathological features. Also, the expression of six-USPs was related to promoter methylation level. Moreover, more than 40% genetic alterations and mutations were discovered in six-USPs. Furthermore, the six-USP expression was correlated with immune infiltration and immune-related prognosis. The functional analysis found that the six-USPs were involved in various biological processes and signaling pathways, such as nucleocytoplasmic transport, choline metabolism in cancer, cell cycle, ErbB signaling pathway, RIG-I-like receptor signaling pathway, TGF-*β* signaling pathway, and TNF signaling pathway. In conclusion, the results showed that six-USPs are potential prognostic biomarkers and can be recruited as possible therapeutic targets of PDAC.

## 1. Introduction

Pancreatic cancer still remains one of the most fatal malignancies in spite of decades of endeavor which is estimated to cause 49830 deaths in 2022 in the US [1]. In China, the estimated deaths are even more than that in the US which will reach 131203 due to the larger population base [2]. Moreover, women and younger individuals are increasingly susceptible to pancreatic cancer which have caused gigantic expenditures worldwide [3, 4]. Pancreatic ductal adenocarcinoma (PDAC) is the most common pathological type which accounts for 90% of pancreatic cancer [5]. Surgery is still the only radical therapy method for PDAC. However, due to the difficulty in early diagnosis, rapid progression, and chemoresistance, the overall survival (OS) of PDAC patients is only about 5% [6]. Moreover, the effect of known targeted therapeutics is not satisfactory. Therefore, novel and effective therapeutic targets are still needed to improve the dismal prognosis of the patients. Posttranslational modifications (PTMs) play essential roles through modifying protein functions/roles and are required for the maintenance of cell viability and biological processes. Therefore, their dysregulation usually leads to disease. Ubiquitination is one of the PTMs which can be involved in various pathological processes including pancreatic pathogenesis [7]. Deubiquitination is the reverse process of ubiquitination by the function of deubiquitinating enzymes (DUBs). DUBs contain seven families, and ubiquitin-specific proteases (USPs) is the major component among them which have been reported to play a pivotal role in different biological processes, such as cell cycle, chromatin remodeling, and DNA damage repair, and also participate in several classical signaling pathways, such as p53, Wnt/*β*-catenin, NF-*κ*B, and TGF-*β* signaling pathways due to its versatile substrates [8]. Moreover, the role of USPs in the initiation and progression of malignancies has been revealed recently. For example, USP4 has been reported to be involved in regulation cell cycle, proliferation, and DNA repair and also participated in progression of various cancers, such as lung cancer, breast cancer, liver cancer, and colorectal cancer. Moreover, the prognostic value of USP4 in cancers has also been confirmed [9]. USP14 has also been revealed its unique role in tumor progression, and several selective USP14 inhibitors have been developed for targeted therapy, such as IU1 and IU1-47 [10]. Although the mechanisms of several USPs in pancreatic pathogenesis have been superficially explored, the clinical significance and biological roles of other USPs in PDAC still remain to be further elucidated.

Through browsing HUGO Gene Nomenclature Committee (HGNC) (http://www.genenames.org), 56 genes constitute the whole USP family. In our present study, six USP family members, namely, USP10, USP14, USP18, USP32, USP33, and USP39 (termed as six-USPs in the following text), were mainly excavated due to limited and robust studies of their role in PDAC. The expression levels of six-USPs between tumor tissues and adjacent tissues or normal pancreatic tissues were compared using different datasets. Then, we systematically analyzed their correlation with clinicopathologic characteristics, promoter methylation in epigenetic regulation, fluorescence localization, gene mutation, immune infiltration, prognostic values, and the Gene Oncology (GO) enrichment analysis and the prediction of Kyoto Encyclopedia of Genes and Genomes (KEGG) pathway using diverse databases and online analytical tools.

## 2. Materials and Methods

### 2.1. GEPIA

GEPIA (http://gepia.cancer-pku.cn) is a web-based tool which can provide diverse function modules or researchers to conduct gene-related studies based on The Cancer Genome Atlas (TCGA) and Genotype-Tissue Expression (GTEx) databases [11]. In our study, we used GEPIA to explore the differential expression of six-USPs between tumor tissues and normal tissues in 33 tumors, and pancreatic adenocarcinoma (PAAD) was included. The relationship between the expression level of six-USPs and PDAC pathological stages was analyzed using the stage plot module.

### 2.2. GEO

The GEO database, developed in 2000, can freely archive and distribute high-throughput data and other functional genomic datasets as an international public repository including several PDAC datasets [12]. Six GEO datasets were used to compare the differential expression between PDAC tissues and normal pancreatic tissues (GSE16515, GSE62165, and GSE101448) or paired adjacent noncancerous tissues (GSE62452, GSE28735, and GSE15471). GSE45757 was recruited to compare the expression level of six-USPs in diverse PDAC cell lines and normal human-pancreatic ductal epithelial cells. Moreover, five other GEO datasets were also utilized for analyzing the relationship between the expression level of six-USPs and clinicopathological features of PDAC. GSE62165 was used to analyze tumor location (head vs. body/tail), and GSE21501 was used to analyze tumor size (T1-T2 vs. T3-T4) and lymphatic metastasis (N0 vs. N1). In addition, GSE62452, GSE19650, and GSE51971 were used to analyze differentiation degree (G1-G2 vs. G3-G4), different precancerous lesions, and cell stemness of tumor, respectively.

### 2.3. UALCAN

The University of ALabama at Birmingham CANcer data analysis Portal (UALCAN) (http://ualcan.path.uab.edu) is an interactive and versatile web portal to perform in-depth analysis based on the TCGA database including relative expression of genes, evaluation of the effect of gene expression and clinicopathological characteristics on survival, and the top related genes in individual cancer types [13]. In this study, UALCAN was used to analyze the correlation between the expression of six-USPs and tumor grades, pathological stages, and TP53 mutation status. Moreover, promoter methylation level was also evaluated between normal and primary tumor tissues, among tumor with different tumor grades, different tumor stages, and different TP53 mutation status.

### 2.4. HPA

The HPA (https://www.proteinatlas.org) database is an online analytical tool which mainly focuses on human protein expression and cellular distribution with basic information of more than 26000 human proteins via three modules, namely, cell, tissue, and pathology. We used the HPA database to obtain immunohistochemical (IHC) staining images of six-USPs and the confocal images of their cellular localization. Besides, the detailed data of survival analysis in HPA pathology module were extracted to perform prognostic analysis of the six-USPs.

### 2.5. KMP

The KMP database (https://kmplot.com/analysis) is a web-based tool focused on survival analysis and can perform univariate and multivariate Cox proportional hazards survival analysis of groups or even subgroups using data generated by genomic, transcriptomic, proteomic, or metabolomic studies [14]. The Kaplan-Meier plotter database was utilized to evaluate the prognostic significance of the expression of six-USPs by OS and recurrence-free survival (RFS). Besides, the subgroup prognostic analysis, including gender, mutation burden, and immune infiltration-related survival, was also conducted. Hazard ratio (HR) and 95% confidence interval (CI) were then calculated automatically according to “Auto select best cutoff.”

### 2.6. Immune Infiltration Analysis of USPs

TIMER (https://cistrome.shinyapps.io/timer) is an online tumor-immune interaction analytical tool which is capable of providing a user-friendly interface for dynamic analysis and association visualization [15]. In our study, the module of gene expression and somatic copy number alterations (SCNA) was used to evaluate the immune infiltration of USPs in PDAC tissues.

### 2.7. LinkedOmics

The LinkedOmics (http://www.linkedomics.org) database is a multiomics database which integrates mass spectrometry-based global proteomics data generated by the Clinical Proteomic Tumor Analysis Consortium and includes multiomics data and clinical data for 32 cancer types and a total of 11158 patients from TCGA project [16]. We recruited the LinkedOmics database for excavating the top 200 positively coexpressed genes with individual USP in PDAC patients using Spearman correlation test.

### 2.8. CBioPortal Database

The cBioPortal database (http://www.cbioportal.org) is an easy and accessible online resource database which can be used to explore, visualize, and comprehensively analyze multidimensional cancer genomic data [17]. In this study, we explored the status of genetic alterations and mutations of six-USPs, and the top 200 coexpressed genes were also identified via the cBioPortal database. We selected “Pancreatic Adenocarcinoma (TCGA, PanCancer Atlas)” (184 samples) for further study. The genomic profiles of “mutations,” “structural variant,” “Putative copy-number alterations from GISTIC,” and “mRNA expression” were selected. “Complete sample (168)” was selected for further investigation.

### 2.9. DAVID

The database for annotation, visualization, and integrated discovery (DAVID) (2021 update) (http://david.ncifcrf.gov) is an approachable bioinformatic system containing a web server and web service for functional annotation and enrichment analyses of gene lists. In the 2021 updated version, the existing annotation types have been updated based on the brand-new DAVID Gene system which increased the taxonomy coverage from 17399 to 55464 [18]. The mutual genes among the top 200 coexpressed genes of six-USPs in both LinkedOmics database and cBioPortal database were input in the DAVID database for functional enrichment using GO and KEGG pathway analysis. The three parts of biological process (BP), cellular component (CC), and molecular function (MF) were included in GO functional enrichment analysis.

### 2.10. Statistical Analysis

Statistical analysis and graphs were conducted and plotted by GraphPad Prism 7.04 (Lajolla, CA, USA). Comparisons between two groups were performed using two-tailed paired or unpaired student *t*-test. As for comparisons of more than two groups, one-way ANOVA with post-hoc Dunnett test or Tukey's test was utilized. *P* value less than 0.05 was considered to be statistically significant.

## 3. Results

### 3.1. Aberrant Expression Levels of USPs in PDAC Patients

**Figure 1:**
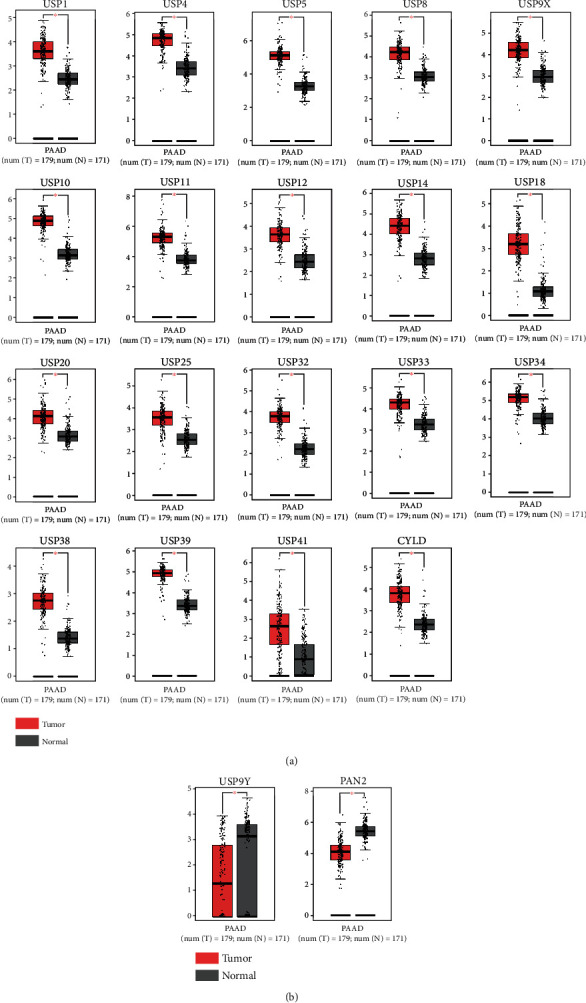
Significantly aberrantly expressed USP family members in pancreatic ductal adenocarcinoma (PDAC) tissues (*n* = 179) and normal pancreatic tissues (*n* = 171). (a) USP family members that are highly expressed in PDAC tissues. (b) USP family members that are lowly expressed in PDAC tissues.

**Figure 2:**
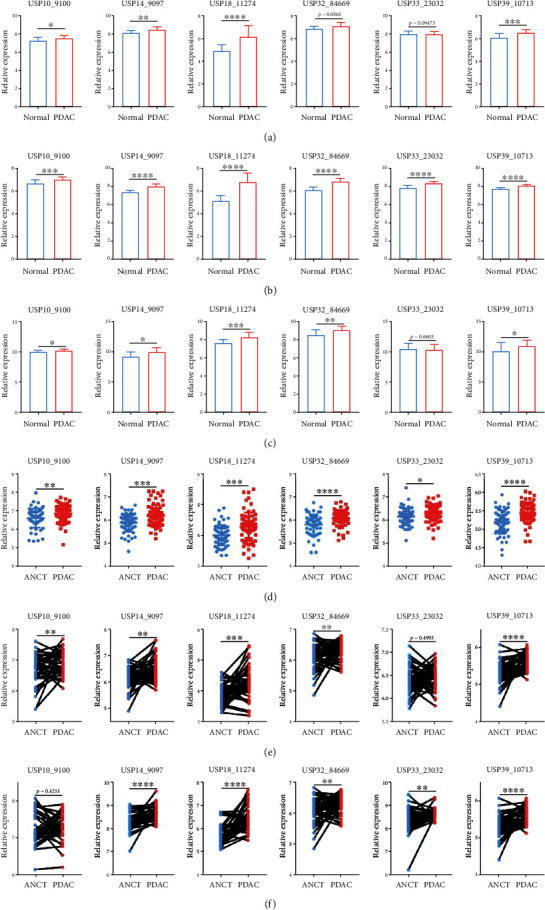
Six datasets were recruited to confirm the differential expression of six-USPs in pancreatic ductal adenocarcinoma (PDAC). (a) The mRNA levels of six-USPs via the comparison between PDAC (*n* = 36) and normal pancreatic tissues (*n* = 16) in GSE16515 dataset. (b)The expression levels of six-USPs in PDAC tissues (*n* = 118) compared with normal tissues (*n* = 13) in GSE62165 dataset. (c) The mRNA levels of six-USPs via the comparison between PDAC (*n* = 18) and normal pancreatic tissues (*n* = 13) in GSE101448 dataset. (d) The expression levels of six-USPs in PDAC tissues (*n* = 69) versus adjacent noncancerous tissues (ANCT) (*n* = 61) in GSE62452 dataset. (e) The transcriptional levels of six-USPs in PDAC tissues compared with ANCT (*n* = 45 pairs) in GSE28735 dataset. (f) The expression levels of six-USPs in PDAC tissues compared with ANCT (*n* = 39) in GSE15471 dataset. The code behind the gene is the gene ID in different GEO datasets. ^∗^*P* < 0.05, ^∗∗^*P* < 0.01, ^∗∗∗^*P* < 0.001, and ^∗∗∗∗^*P* < 0.0001.

Firstly, the GEPIA database, an online gene comprehensive analysis tool which is based on TCGA and GTEx datasets, was used to identify the differential mRNA expression of USPs between PDAC tissues (*N* = 179) and normal pancreatic tissues (*N* = 171). Among all 56 genes in the USP family, 21 of them exhibited significantly differential expression between PDAC tissues and normal pancreatic tissues. Except for USP9Y and CYLD, the rest of 19 USPs were highly expressed in PDAC tissues which was presented in Figure 1. The rest of USPs which showed no significantly differential expression were presented in Supplementary Figure S1. Then, we conducted an in-depth literature investigation. Among the 21 USPs, limited studies were reported between USP10, USP14, USP18, USP32, USP33 and USP39, and PDAC. Therefore, the six-USPs further underwent in-depth exploration in the present study. We then investigated the transcriptional levels of six-USPs in other 32 tumors. The result showed that all the rest four USPs were aberrantly expressed in several other tumors with USP32 lower expression in testicular germ cell tumors and USP33 higher expression in thymoma (Supplementary Figure S2). Besides, six GEO datasets were recruited to further confirm the discrepancy in the expression of above six-USPs between PDAC and normal pancreatic tissues (GSE16515, GSE62165, and GSE101448) or adjacent noncancerous tissues (GSE62452, GSE28735, and GSE15471). The result indicated that, generally, the expression levels of six-USPs in PDAC tissues were significantly elevated, although USP33 did not show significantly differential expression in three datasets and USP10 and USP32 in one dataset (Figures 2(a)–2(f)).

### 3.2. The Relationship between Six-USPs Expression and the Clinicopathological Characteristics of PDAC Patients

**Figure 3:**
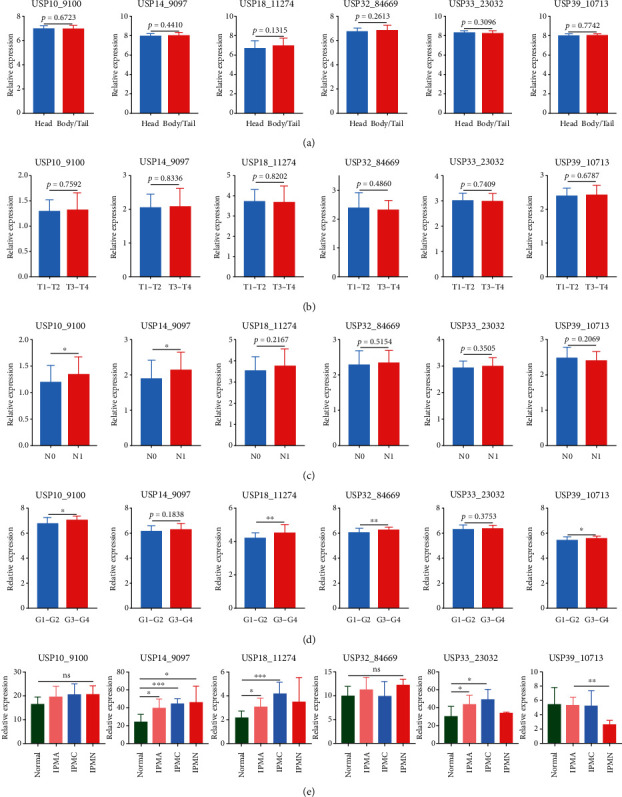
Correlation between six-USP expression and the clinicopathological features of pancreatic ductal adenocarcinoma (PDAC) patients in GEO datasets. (a) The comparison between head (*n* = 93) and body/tail (*n* = 25) of PDAC from GSE62165. (b) The expression levels of six-USPs via the comparison between T1-T2 (*n* = 18) and T3-T4 (*n* = 80) in GSE21501 dataset. (c) The expression levels of six- USPs in PDAC patients with/without lymphatic metastasis (N1/N0, 73 vs. 28) in GSE21501 dataset. (d) The mRNA levels of six-USPs in different differentiated degrees of PDAC (G1-G2 vs. G3-G4, 37 vs. 31) using GSE62452 dataset. (e) The transcriptional levels of six-USPs in different pancreatic precancerous lesions compared with normal pancreatic tissues using GSE19650 dataset. Normal (*n* = 7), IPMA (*n* = 6), IPMC (*n* = 6), and IPMN (*n* = 3). The code behind the gene is the gene ID in different GEO datasets. ^∗^*P* < 0.05, ^∗∗^*P* < 0.01, and ^∗∗∗^*P* < 0.001. n.s.: not significant difference.

**Figure 4:**
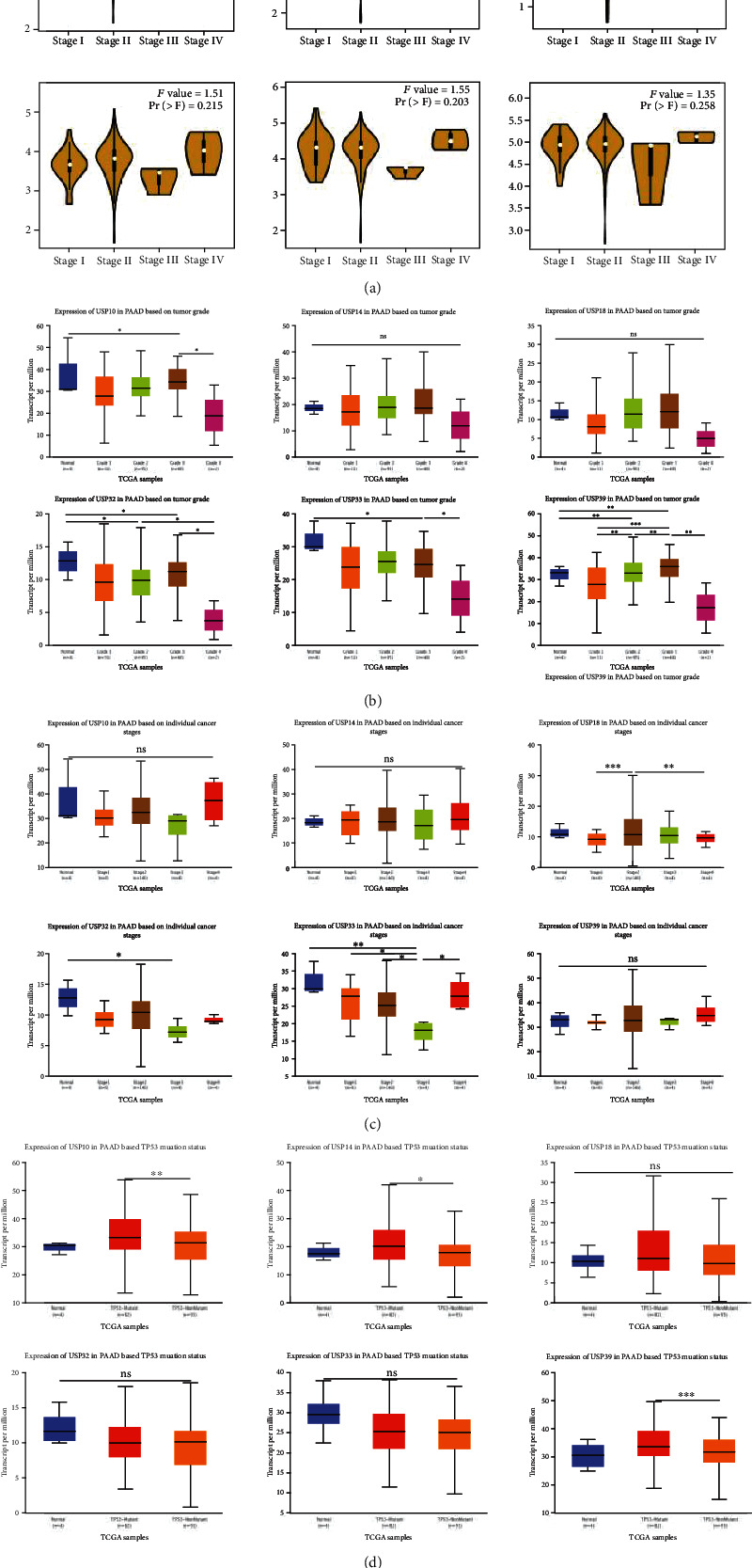
Correlation between six-USP expression and the clinicopathological characteristics and P53 mutation in pancreatic ductal adenocarcinoma (PDAC) patients via GEPIA and UALCAN databases. (a) The expression levels of six-USPs in different pathological stages using GEPIA. (b) The transcriptional levels of six-USPs in different differentiation degrees of PDAC. (c) The expression levels of six-USPs in different pathological stages. (d) The expression levels of six-USPs in PDAC patients with/without P53 mutation. ^∗^*P* < 0.05, ^∗∗^*P* < 0.01, and ^∗∗∗^*P* < 0.001. n.s.: not significant difference.

Subsequently, we explored the potential clinical value of six-USPs in PDAC patients via analyzing three GEO datasets (GSE62165, GSE21501, and GSE62452), GEPIA, and UALCAN databases. The result from GSE62165 and GSE21501 indicated that the expression levels of six-USPs were not related to tumor location (*N* = 118) and tumor size (*N* = 98) (Figures 3(a) and 3(b)), while GSE21501 data showed that USP10 and USP14 were robustly associated with lymphatic metastasis (*N* = 101) (Figure 3(c)). GSE62452 revealed that high USP10, USP18, USP32, and USP39 expressions were linked to poor differentiation of PDAC (Figure 3(d)). However, the result of GEPIA did not show significant relationship between six-USPs expression and PDAC pathological staging (Figure 4(a)). We then recruited UALCAN for further analysis in regard to tumor differentiation and clinical staging based on TCGA database. The result in tumor differentiation analysis indicated that only the expression level of USP39 was tightly correlated with tumor differentiation in which the high USP39 expression is related to poor tumor differentiation in general. Although the expression levels of USP10, USP32, and USP33 were also significantly related to tumor differentiation to some extent, the result may be unreliable due to the small sample size (Figure 4(b)). Moreover, the expression levels of six-USPs were not correlated with tumor clinical staging in general which was similar to the result of GEPIA (Figure 4(c)). TP53 is one of the four major driver genes of PDAC whose mutation plays a vital role in its initiation and progression [19]. We therefore explored the relationship between the expression levels of six-USPs and TP53 mutation status. USP10, USP14, and USP39 were found higher expression in the TP53-mutant group than that in the TP53-nonmutant group while the expression of USP18, USP32, and USP 33 showed no significant difference between the two groups (Figure 4(d)).

PDAC can gradually occur and undergo multiprocesses from its precancerous lesions, such as pancreatic intraepithelial neoplasia [20]. We hence compared the expression levels of six-USPs in several precancerous lesions, including intraductal papillary-mucinous adenoma (IPMA), intraductal papillary-mucinous carcinoma (IPMC), and intraductal papillary-mucinous neoplasm (IPMN) via analyzing GSE19650 dataset. The results demonstrated that the expression of USP14 in three precancerous lesions (IPMA, IPMC, and IPMN) was higher than that in normal pancreatic tissues. And the expression of USP14 was the highest in IPMN among the three precancerous lesions (Figure 3(e)). Moreover, USP18 and USP33 expressions in IPMA and IPMC were higher than that in normal pancreatic tissues, while IPMN did not show significantly differential expression compared to normal pancreatic tissues (Figure 3(e)) and the USP39 expression was downregulated in IPMN compared to that in IPMA (Figure 3(e)). However, USP10 and USP32 did not show significantly differential expression between precancerous lesions and normal pancreatic tissues.

### 3.3. The Relationship between Promoter Methylation Levels of Six-USPs and Clinicopathological Features

Promoter methylation is one of the common epigenetic modifications which influences transcriptional regulation of genes in various cancers, PDAC included, and can be used as therapeutic targets [21]. The levels of promoter methylation of six-USPs in PDAC and normal pancreatic tissues were investigated in our study using the UALCAN database. The result indicated that USP14 and USP39 had higher levels of promoter methylation in PDAC tissues than that in normal pancreatic tissues (Supplementary Figure S3A). As for the relationship between clinicopathological features and promoter methylation, the promoter methylation levels of USP14, USP18, and USP39 were significantly related to PDAC differentiation (Supplementary Figure S3B), while the promoter methylation levels of USP14 and USP39 were significantly correlated with PDAC clinical staging (Supplementary Figure S4A). Notably, the promoter methylation levels of USP33 presented prominent positive correlation with tumor differentiation except for grade 4 because of the relatively small sample capacity. Moreover, the promoters' methylation levels of USP10, USP18, and USP32 were lower in the TP53-mutant group than that in the TP53-nonmutant group, while the rest of USPs did not show the similar differences (Supplementary Figure S4B).

### 3.4. The Tissues Protein and Cell Line Expression, Cellular Localization, and Cell Stemness of Six-USPs in PDAC

**Figure 5:**
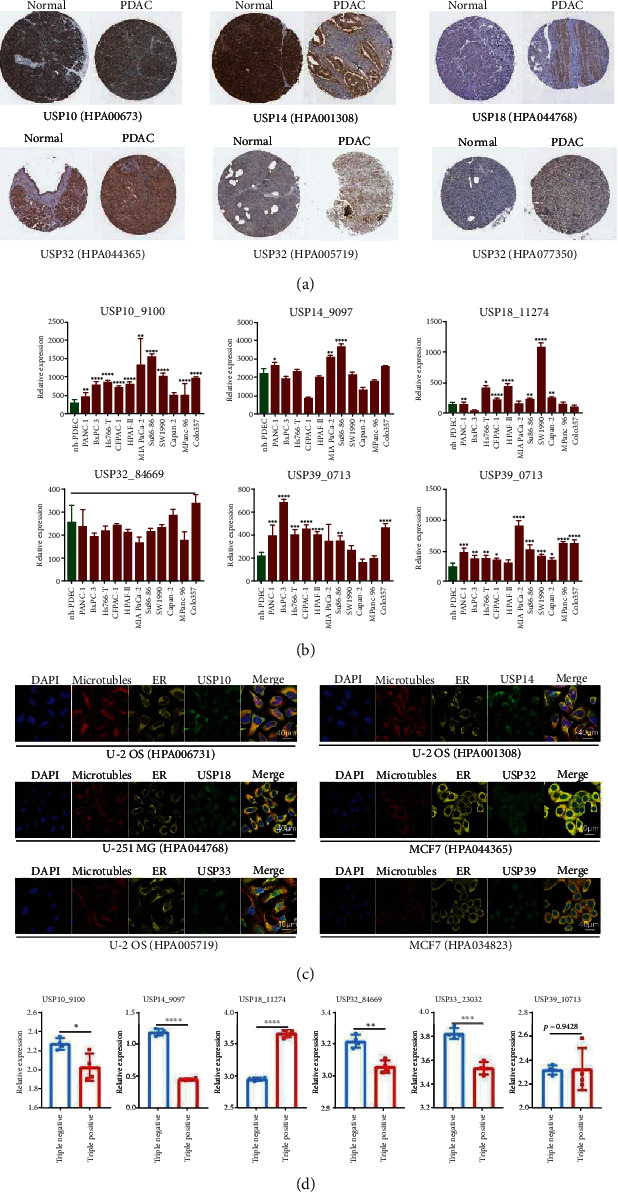
The protein expression, cell lines, cellular localization, and cell stemness of six-USPs in pancreatic ductal adenocarcinoma (PDAC). (a) The protein expression of six-USPs in PDAC and normal pancreatic tissues using immunohistochemistry (IHC) in the HPA database. The code behind the gene is the related primary antibody in HPA. (b) The expression levels of six-USPs in human normal pancreatic ductal epithelial cells (hn-PDEC) and multiple PDAC cell lines using GSE45757 dataset. (c) The confocal images of cellular localization of six-USPs in different types of cells using the HPA database. The code behind the cell line is the related primary antibody in HPA. ER: endoplasmic reticulum. (d) The expression levels of six-USPs in triple-positive group (*n* = 4) and triple-negative group (*n* = 4), which were classified by three key cancer stem cell markers, CD44, CD133, and EpCAM, using GSE51971 dataset. The code behind the gene is the gene ID. ^∗^*P* < 0.05, ^∗∗^*P* < 0.01, ^∗∗∗^*P* < 0.001, and ^∗∗∗∗^*P* < 0.0001.

Then, the protein expression in PDAC and normal pancreatic tissues of six-USPs was detected through the HPA database. As was shown in Figure 5(a), only the protein expression of USP33 and USP39 was significantly elevated in PDAC tissues compared to corresponding normal pancreatic tissues. However, the expression level of USP14 was significantly higher in normal pancreatic tissues than that in PDAC tissues. Subsequently, GSE45747 was recruited for identifying six-USPs expression in human normal pancreatic ductal epithelial cells (hn-PDEC) and several PDAC cell lines, including PANC-1, BxPC-3, Hs766-T, CFPAC-1, HPAF-II, MIA PaCa-2, Su86.86, SW1990, Capan-2, MPanc-96, and Colo357. The result indicated that the expression levels of USP10, USP18, USP33, and USP39 were significantly higher in most included PDAC cell lines than hn-PDEC while USP14 only highly expressed in PANC-1, MIA PaCa-2, and Su86.86 cell lines. However, USP32 did not present significantly differential expression among all these cell lines (Figure 5(b)). The intracellular functions of proteins usually depend on its cellular localization. Therefore, the intracellular localizations of six-USPs were also explored via confocal fluorescence imaging of HPA database. The result revealed that USP10 was detected in cytosol and nucleoplasm while USP14 was detected in plasma membrane and cytosol. USP18 was detected in cytosol and intracellular vesicles, and USP32 was localized in Golgi apparatus and cytosol. USP33 was distributed in nucleoplasm and Golgi apparatus, while USP39 was localized in nucleoplasm (Figure 5(c)). GSE51971 dataset contains the information about MIA PaCa-2 cell line that was sorted by fluorescence activated cell sorting using 3 markers: CD44, CD133, and EpCAM. And the PDAC cell line was divided into triple-negative group and triple-positive group to show the cell stemness. We used GSE51971 dataset to explore the correlation between six-USPs expression and PDAC cell stemness. The result demonstrated that the expression levels of USP10, USP14, USP32, and USP33 were upregulated in the triple-negative cell subgroup while the USP18 expression was higher in the triple-positive subgroup than that in the triple-negative subgroup (Figure 5(d)). The aforementioned result indicated that six-USPs may play an important role in PDAC initiation.

### 3.5. The Prognostic Value of Six-USPs in PDAC Patients

**Figure 6:**
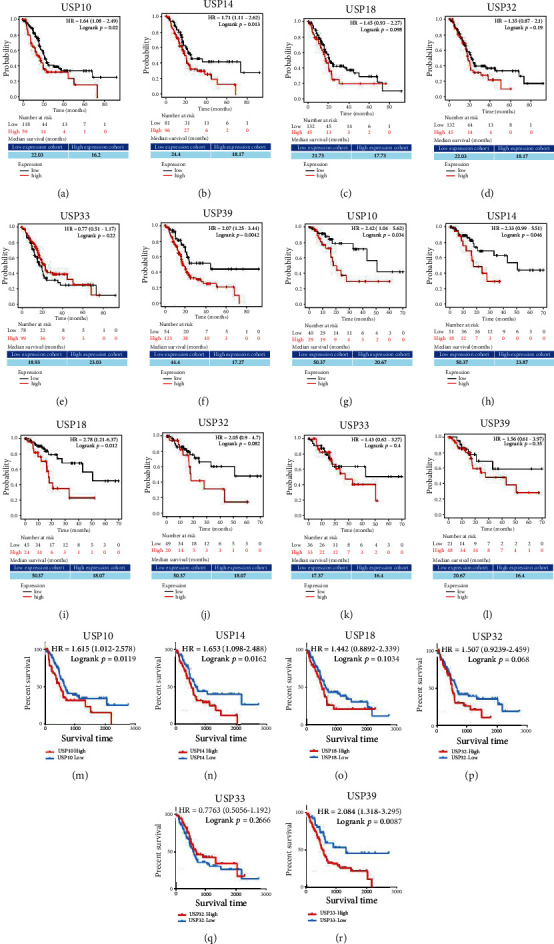
The prognostic analysis of the six-USPs in pancreatic ductal adenocarcinoma (PDAC) using Kaplan-Meier plotter. (a–f) The overall survival of the six-USPs in PDAC patients (*N* = 177). (g–l) The recurrence-free survival of the six-USPs in PDAC patients (*N* = 69). (m–r) The overall survival of the six-USPs in PDAC patients using the HPA database (*N* = 176).

The genes which show differential expression between tumor and normal tissues may possess significantly prognostic value in cancer patients [22]. In order to identify the prognostic significance of six-USPs, KMP and HPA databases were utilized for survival analysis. The result of the KMP database indicated that high expression levels of USP10, USP14, and USP39 were significantly related to a poor OS in PDAC patients while USP18 expression showed a marginal significance (0.05 < *P* < 0.1) and USP32 and USP33 were not significant associated with the OS of PDAC patients. Specifically, USP39 had the highest HR in OS at 2.07 which indicated that the risk of death in the USP39 high-expression group was 2.07 times higher than that of the USP39 low-expression group. The median OS time of USP39 high-expression group and low-expression group was 17.27 and 44.4 months, respectively (Figures 6(a)–6(f)). As for RFS, higher USP10, USP14, and USP18 expressions could predict a worse RFS of PDAC patients while the rest three USPs could not. In detail, USP18 had the highest HR in RFS at 2.78 which the risk of relapse in the USP18 high-expression group was 2.78 times higher than that of the USP18 low-expression group. The median RFS time of USP18 high-expression group and low-expression group was 18.07 and 50.37 months, respectively (Figures 6(g)–6(l)). Moreover, high USP10, USP14, and USP39 expressions were found to significantly be associated with a poor OS of PDAC patients, and the USP32 expression showed a marginal significance (0.05 < *P* < 0.1) while USP18 and USP33 did not exhibit a remarkable prognostic value in PDAC patients via analysis of the HPA database (Figures 6(m)–6(r)). The aforementioned result indicated that the four USPs were likely to be used as potential prognostic biomarkers in PDAC patients except for USP32 and USP33.

### 3.6. The Immune Infiltration Level and Immune-Related Prognosis of Six-USPs in PDAC

**Figure 7:**
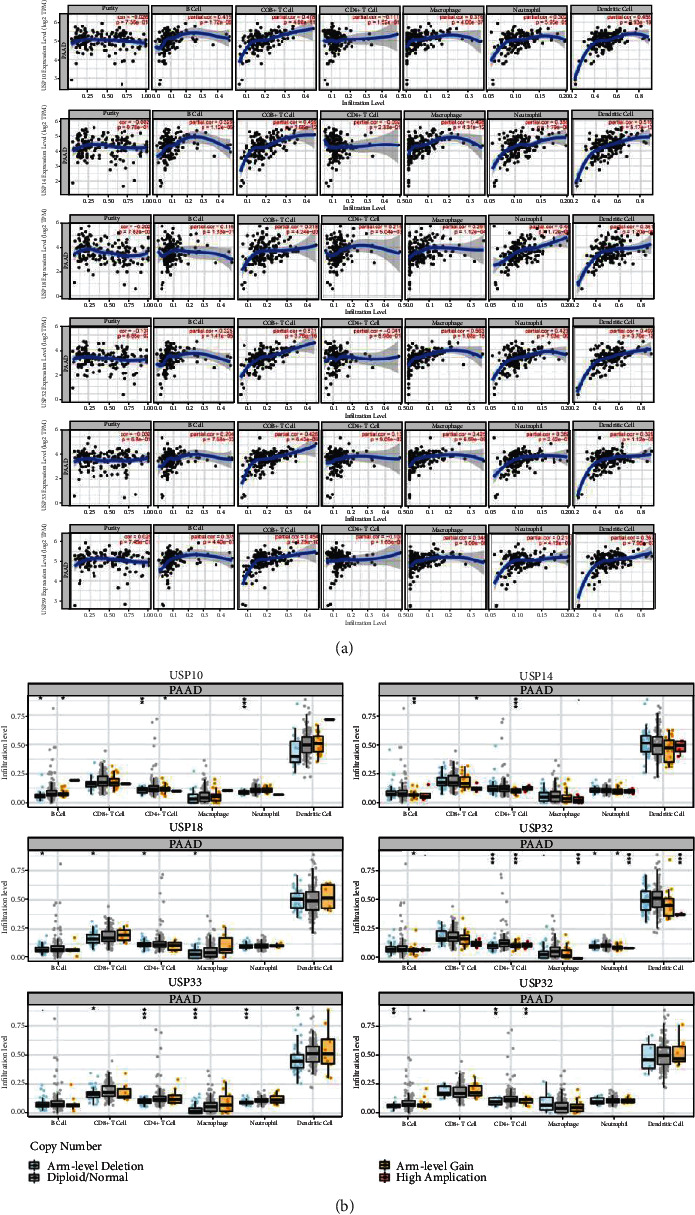
Relationship between six-USPs and immune infiltration in PDAC (TIMER, Spearman correlation). (a) The association between six-USP expression and the infiltration of immune cells in PDAC. (b) The role of SCNA in six-USPs and immune cell infiltration in PDAC. The immune cells included B cells, CD8+ T cells, CD4+ T cells, macrophages, neutrophils, and dendritic cells. ^∗^*P* < 0.05, ^∗∗^*P* < 0.01, and ^∗∗∗^*P* < 0.001.

The relationship between six-USP expression and various immune cell infiltration in PDAC was deeply studied using TIMER database. The expression levels of USP10, USP14, USP32, USP33, and USP39 was positively significantly correlated with the infiltration of B cells while all six-USP expressions were positively significantly related to the infiltration of CD8+ T cells, macrophage, and dendritic cells. However, only USP18 expression was positively significantly correlated with CD4+ T cell infiltration (Figure 7(a)). In addition, the SCNAs of the six-USP expression were evaluated using SCNA module of TIMER. The result revealed that the SCNA of all six-USPs except for USP33 was significantly related to B cell infiltration level, and the SCNA of USP10, USP18, and USP33 was significantly correlated with CD8+ T cell infiltration level. Moreover, the SCNAs of all six-USPs were significantly associated with CD4+ T cell infiltration level. And the SCNA of USP18, USP32, and USP33 was significantly related to macrophage infiltration while USP10, USP32, and USP33 SCNAs were significantly correlated with neutrophil infiltration in PDAC. However, only USP32 and USP33 presented significant correlation with dendritic cell infiltration (Figure 7(b)).

Given the significant correlation between six-USP expression and immune cell infiltration of PDAC, the prognosis of immune subgroups of PDAC patients was hence explored using the Kaplan-Meier plotter database. The analytic data obtained from the Kaplan-Meier plotter database indicated that USP10 and USP39 were the negative prognostic factors in the B cell-enriched subgroup while USP10 was the negative prognostic factors in the CD8+ T cell-decreased subgroup. As for macrophages, USP14 and USP18 were the negative prognostic factors in the macrophage-enriched subgroup while USP10 was the negative prognostic factor in the macrophage-decreased subgroup. Besides, only the USP10 expression showed a robustly negative correlation in the subgroup of regulatory T cell-decreased PDAC patients (Supplementary Figure 5). Generally, the six-USPs were inclined to be the negative prognosis factors in the immunosuppressed state of PDAC. Whereafter, the prognostic analysis of the subgroups of different genders and mutation burdens were also detected. The results demonstrated that USP10, USP14, and USP18 were the negative prognostic factors in the male group. And USP10 and USP18 were the negative prognostic factors in the high mutation burden subgroup while USP14 was the negative prognostic factor in the low mutation burden subgroup (Supplementary Figure 5). Additionally, the data subgroup prognostic analysis of DFS was also exhibited in Supplementary Figure 6.

### 3.7. The Genetic Alteration and Mutation Information of Six-USPs in PDAC

**Figure 8:**
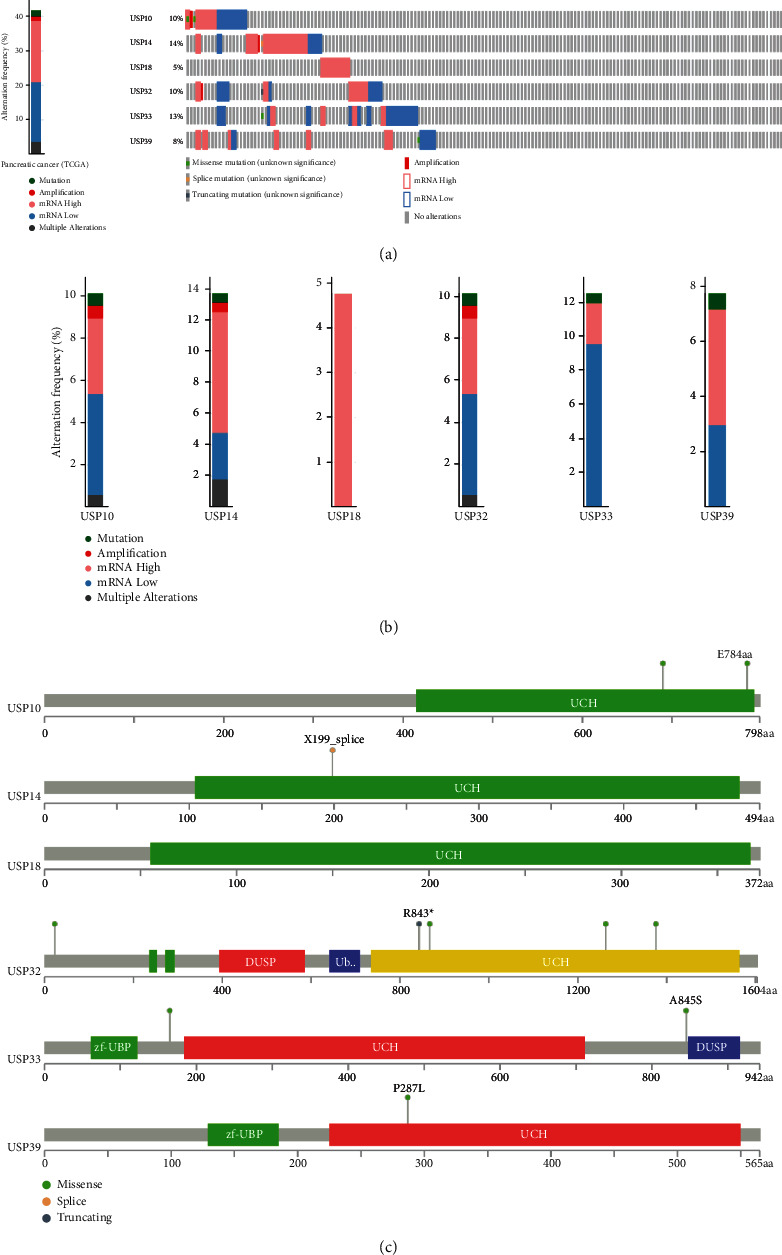
The mutation information of six-USPs in pancreatic ductal adenocarcinoma (cBioPortal). (a) Alteration frequency of six-USPs at the overall and individual levels. (b) The bar graph of gene alteration frequency of six-USPs. (c) Schematic representation of gene mutation sites of six-USPs on the coding strand.

Genetic alteration and mutation play a decisive role in malignancy transition from normal pancreatic duct epithelium to PDAC [23]. Therefore, the genetic alteration and mutation states of six-USPs in PDAC were further elucidated via cBioPortal database (TCGA, PanCancer Atlas). The result revealed that 70 of 168 patients were presented target gene alteration to varying degrees with the percentage of 41.67% (Figure 8(a)). Concretely, USP14 showed the highest frequency of alteration (23 of 168 cases, 13.69%), with mRNA upregulation in 13 cases (7.74%), mRNA downregulation in 5 cases (2.98%), multiple alterations in 3 cases (1.79%), and mutation and amplification in 1 case (0.6%), respectively (Figures 8(a) and 8(b)). USP33 followed closely by USP14 with the frequency of alteration of 12.5% (21 cases). The major genetic alterations were mRNA downregulation (16 of 168 cases, 9.52%), mRNA upregulation (4 of 168 cases, 2.38%), and mutation (1 of 168 cases, 0.6%) (Figures 8(a) and 8(b)). Other genetic alteration contained USP10 and USP32 (both 17 of 168 cases, 10.12%), USP39 (13 of 168 cases, 7.74%), and USP18 (8 of 168 cases, 4.76%). The detailed constitutes and their respective percentages of the genetic alteration of six-USPs were shown in Figure 8(b). Most USPs we included showed high mRNA expression mutation except for USP33 whose mRNA downregulation occupied a major proportion. Interestingly, USP18 only showed high mRNA expression in 5 (4.76%) cases without other genetic alterations. Subsequently, we used “mutations” module to retrieve mutation information of six-USPs. The overall somatic mutation frequency remained extremely low. The frequency of USP14, USP32, USP33, and USP39 was 0.6% while USP10 was 1.2%. And USP18 even did not occur somatic mutation.  Figure 8(c) displays the detailed mutation sites in six-USP DNA sequences. The green dots demonstrated the missense mutation sites, and the yellow dots indicated the splice mutation sites while the grey dots represented the truncating mutation sites. The results implied that the six-USPs have genetic stability to be potential biomarkers of PDAC, especially USP18.

### 3.8. Analysis of Functions and Pathways for Six-USPs in PDAC

**Figure 9:**
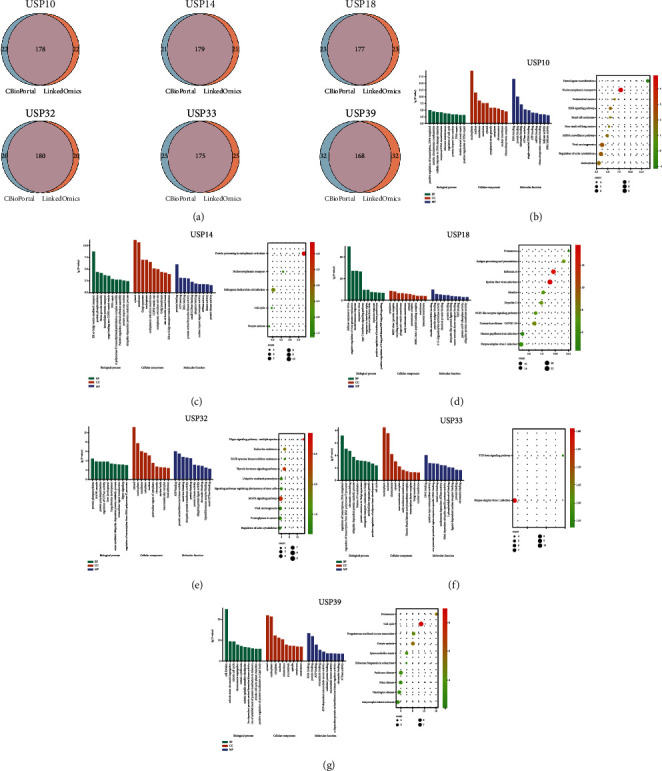
Systematic bioinformatic analysis of six-USPs and individual coexpressed genes in pancreatic ductal adenocarcinoma. (a) Intersection of top 200 positively coexpressed genes analyzed by the LinkedOmics and the cBioPortal databases (Spearman correlation). (b–g) The Gene Ontology enrichment analysis and Kyoto Encyclopedia of Genes and Genomes pathway prediction of six-USPs were conducted. BP: biological processes; CC: cellular components; MF: molecular function.

To deeply explore the potential mechanisms and pathways influenced of six-USPs in PDAC, we primarily screened out the top 200 positively coexpressed genes via cBioPortal and LinkedOmics, respectively, and acquired the intersected genes of the six-USPs (Figure 9(a)). The potential functions and pathways of the six-USPs were generated by using the shared genes to perform GO and KEGG enrichment analysis. The three parts of BP, CC, and MF were contained in GO enrichment analysis.

For USP10, BP terms included positive regulation of transcription, DNA-templated, cellular response to DNA damage stimulus, cell cycle regulation, and DNA repair. CC terms were implicated in nucleoplasm, nucleus, and membrane. MF terms contained RNA binding, protein binding, chromatin binding, and single-stranded DNA binding. The KEGG pathways indicated that USP10 was related to nucleocytoplasmic transport, mRNA surveillance pathway, and ErbB signaling pathway (Figure 9(b)). For USP14, BP terms included ER to Golgi vesicle-mediated transport, protein phosphorylation, COPII vesicle coating, intracellular protein transport, and DNA repair. CC terms were involved in COPII vesicle coating, Golgi apparatus, and endoplasmic reticulum membrane. MF terms included protein binding, ATP binding, and protein serine/threonine/tyrosine kinase activity. The KEGG pathways indicated that USP14 was related to protein processing in endoplasmic reticulum, nucleocytoplasmic transport, and cell cycle (Figure 9(c)). For USP18, BP terms were implicated in type I interferon signaling pathway, response to virus, innate immune response, and negative regulation of viral genome replication. CC terms were involved in MHC class I protein complex, phagocytic vesicle membrane, and proteasome complex. MF terms included 2′-5′-oligoadenylate synthetase activity, TAP binding, and ubiquitin-like protein ligase binding. The KEGG pathways demonstrated that USP18 was related to antigen processing and presentation, COVID-19, and NOD-like receptor signaling pathway (Figure 9(d)). For USP32, BP terms included activation of MAPK cascade, liver development, protein phosphorylation, protein polyubiquitination, and intracellular signal transduction. CC terms were implicated in nuclear body, focal adhesion, and perinuclear region of cytoplasm. MF terms included in ATP binding, ubiquitin-protein transferase activity, and ubiquitin-protein ligase activity. The KEGG pathways indicated that USP32 was associated with Hippo signaling pathway, MAPK signaling pathway, viral carcinogenesis, and ubiquitin-mediated proteolysis (Figure 9(e)). For USP33, BP terms contained regulation of transcription, DNA-templated, regulation of transcription from RNA polymerase II promoter, chromatin remodeling, multicellular organism growth, and cell cycle. CC terms were implicated in transcription factor complex, piccolo NuA4 histone acetyltransferase complex, and macromolecular complex. MF terms included cysteine-type endopeptidase activity, RNA polymerase II core promoter proximal region sequence-specific DNA binding, and thiol-dependent ubiquitin-specific protease activity. The KEGG pathways indicated that USP33 was related to Herpes simplex virus 1 infection and TGF-beta signaling pathway (Figure 9(f)). For USP39, BP terms were involved in cell division, mitotic sister chromatid segregation, mitotic cell cycle, and mitotic cell cycle phase transition. MF terms implied microtubule binding, ATP-dependent microtubule motor activity, and cyclin-dependent protein serine/threonine kinase regulator activity. The KEGG pathways demonstrated that USP39 was correlated to proteasome, cell cycle, and oocyte meiosis (Figure 9(g)).

## 4. Discussion

In the present study, we systematically analyzed the expression status, clinicopathological features, prognostic value, and functions of USP family genes, particularly the six-USPs (USP10, USP14, USP18, USP32, USP33, and USP39). Initially, using the GEPIA database, we found the aberrant expression of several USPs between PDAC and normal pancreatic tissues and identified six-USPs which were rarely reported in previous studies. Subsequently, several datasets of the GEO database were recruited to further confirm the differential expression of the six-USPs between PDAC and normal pancreatic tissues or ANCT which all the analytical data of these datasets mutually obtained the similar results and significant differential expression, although the sample capacity of some datasets was relatively small. Moreover, the relationship between the expression of six-USPs or the methylation levels of six-USPs and clinicopathological features was further evaluated via GEO datasets and GEPIA and UALCAN databases. Then, the protein expression in tissues, cell lines and cellular localization, and cell stemness were investigated using HPA database and GEO datasets. The USP-related immune infiltration, genetic alteration, and mutation were further estimated in PDAC tissues using TIMER and cBioPortal databases. Ultimately, we explored the potential functions and pathways of six-USPs using positively coexpressed genes by GO and KEGG enrichment analyses. Although the roles of some members of six-USPs in PDAC initiation and progression have been previously reported, these studies remained relatively superficial and comprehensive, and in-depth analysis of the potential mechanisms of six-USPs in PDAC has yet to be performed [24–27]. Our study comprehensively analyzed the expression status, clinicopathological characteristic relationship, prognostic significance, immune infiltration and functions, and pathway enrichment analysis of six USP family members (USP10, USP14, USP18, USP32, USP33, and USP39) in PDAC for the first time. We hope that our bioinformatic analysis will update the existing knowledge about the six-USPs and may stimulate the enthusiasm for further basic experiments of the six-USPs as well as explore their potential prognostic and target features in PDAC.

### 4.1. USP10

USP10 contains an Ataxin2C domain and a USP domain, and USP domain consists of a catalytic site, protein-protein interaction sites, and localization domains while the function of the Ataxin2C domain remains unknown [28]. Numerous studies have reported the diverse roles of USP10 in tumor progression. The study of Li et al. revealed that USP10 was upregulated in colorectal cancer tissues compared to adjacent normal tissues and also could promote the proliferation and metastatic ability of colorectal cancer cells and the polarization of tumor-associated macrophages through interacting with and deubiquitinating NACHT, LRR, and PYD domain-containing protein 7 at its K379 lysine acceptor site which promoted downstream NF-*κ*B signaling pathway nuclear translocation and activation of C-C motif chemokine ligand 2 transcription [29]. In NSCLC, histone deacetylases 7 (HDAC7) showed an elevated expression in tumor tissues and high HDAC7 expression related to the poor prognosis. In terms of mechanism, HDAC7 could interact with *β*-catenin and enhance its nucleus translocation via decreasing *β*-catenin acetylation level at Lys49 and phosphorylation level at Ser45 to promote fibroblast growth factor 18 expression which induced the malignant biological behaviors of NSCLC, and the HDAC7 expression could be further stabilized by USP10. The similar mechanism could also be found in the study of HDAC6 [30, 31]. Besides, several articles have indicated the roles of USP10 in pancreatic cancer. The study of Liu et al. showed that USP10 could stabilize Yes1-associated transcriptional regulator expression which, whereafter enhanced cysteine rich angiogenic inducer 61 expression to promote immune escape and maintain cell proliferation and metastasis ability via the upregulation of PD-L1 and galectin-9 [24]. Recently, a bioinformatic study comprehensively analyzed the prognostic value and immune infiltration of USP10 in pan-cancer, especially in PDAC and hepatocellular carcinoma [32]. In this study, we found that USP10 was highly expressed in PDAC tissues, and high USP10 expression also indicated a poor prognosis. The expression of USP10 was related to several clinicopathological features of PDAC patients, such as tumor grade, tumor differentiation, TP53 mutation, promoter methylation, and also immune infiltration.

### 4.2. USP14

USP14 is also a well-known tumor promoter similar to USP10 which contains USP domain in C-terminal and ubiquitin-like (UBL) domain in N-terminal to regulate proteasomal activity, and BL1 and BL2 domains are also key factors for USP14 activity [10]. In hepatocellular carcinoma, USP14 was found highly expressed in HCC tissues and was a negative prognostic biomarker in HCC. In-depth study indicated that USP14 could stabilize and transactivate HIF-1*α* to induce its nuclear transfer and promote the ability of cell proliferation, invasion, and migration and vascular mimicry formation [33]. Several studies have revealed the versatile roles of USP14 in lung cancer. USP14 discovered accumulated expression in NSCLC which also showed prognostic value. USP14 inhibition could hamper cell proliferation and cause G2/M phase retardation. Besides, downregulation of USP14 also induced autophagy via ubiquitinated proteins/ER stress/unfolded protein response (UPR) axis by activation of c-Jun N-terminal kinase 1 (JNK1) [34, 35]. Due to the potential of therapeutic target in various cancers, several USP14 inhibitors have been gradually developed to confirm their effect. The review of Wang et al. summarized the development and optimization of USP14 inhibitors from the nonselective b-AP15 and VLX1570 to selective CID43013232 and CID 112370349 [10]. As for the relationship between USP14 and PDAC, the existent study is few and superficial. USP14 found higher expression in PDAC tissues than that in normal pancreatic tissues and also associated the prognosis of PDAC patients. Moreover, the USP14 overexpression could enhance the proliferation, migration, and invasion and also reduce apoptosis of PDAC cells via regulating the expression of cyclin D1, PCNA, and E-cadherin [25]. Our study was basically consistent with the results of the aforementioned articles. In addition, USP14 was also found related to precancerous lesions of PDAC, such as IPMA, IPMC, and IPMN. Therefore, USP14 was expected to be a biomarker for early diagnosis of PDAC. Moreover, the KEGG pathway enrichment analysis indicated that USP14 was related to cell cycle which may be involved in the progression of PDAC.

### 4.3. USP18

USP18, an IFN-stimulated gene 15 (ISG15) DUB, which contains *α*-helical thumb domain at the N-terminus, the C-terminal palm domain, and the finger domain, has been reported to occupy essential position in various physiological and pathophysiological processes, especially in cancers. The study of Liu et al. indicated that USP18 could enhance fatty acid metabolism of lung cancer cells via augmentation of adipose triglyceride lipase (ATGL) and uncoupling protein 1 (UCP1) expression which further promote cell proliferation [36]. Except for regulation of fat acid metabolism, USP18 has also been reported to be involved in antitumor immune response. USP18 showed low expression in extranodal diffuse large B cell lymphoma (EN DLBCL) which was one of the vital immune-related genes. Bioinformatic analysis revealed that USP18 was positively correlated to immune infiltration of activated dendritic cells (aDCs) which implied that USP18 might be involved in DC-modulating immune response [37]. Moreover, given USP18 is a major negative regulator of the IFN signaling pathway, chronic myeloid leukemia (CML) USP18 deficiency cells were found to be more antigenic, driving increased activation of cytotoxic T lymphocyte (CTLs) and were also more susceptible to irradiation [38]. USP18 are also related to the progression of virus-induced cancers. The high expression of USP18 was found in cervical cancer cells which enhanced cell proliferation and hampered apoptosis via PI3K/Akt signaling pathway [39]. In PDAC, USP18 was considered as a tumor promoter and possess significant prognostic value whose overexpression could promote cell proliferation through removing K48-linked ubiquitin from Notch-1 and activating Notch-1/c-Myc signaling pathway [26]. Our study further confirmed the results of the aforementioned studies. Besides, we identified the role of USP18 in immune response of PDAC. The present study indicated that USP18 was significantly related to the infiltration of neutrophils and dendritic cells. The GO enrichment analysis showed that USP18 was associated with several immune or antiviral immune response processes, such as type I interferon signaling pathway, innate immune response, and response to virus, and the KEGG enrichment analysis indicated that USP18 was related to antigen processing and presentation, HPV infection, and herpes simplex virus 1 infection. Therefore, the in-depth mechanisms between USP18 and immune response of PDAC deserved to be explored.

### 4.4. USP32

Through analyzing the public epithelial ovarian cancer (EOC) microarray datasets and the in vivo shRNA screening dataset of Nakae et al. for novel therapeutic targets, USP32 was among the top-ranked genes which expressed in primary ovarian cancer and also deemed as a negative prognostic biomarker. Moreover, USP32 could promote proliferation and epithelial mesenchymal transition abilities of EOC cells via stabilizing farnesyl-diphosphate farnesyltransferase 1 (FDFT1) and regulating mevalonate pathway [40]. The study of Dou et al. indicated that the high USP32 expression was significantly related to a high T stage and poor prognosis in gastric cancer, and USP32 could stabilize SMAD2 protein level to promote cell growth, motility, and chemoresistance to cisplatin [41]. In the present study, we found that the USP32 expression in PDAC tissues was significantly higher than that in normal pancreatic tissues and also significantly associated with tumor grade, tumor stage, and immune infiltration. However, USP32 may not be a potential prognostic biomarker in PDAC given no significant survival difference between USP32-high group and USP32-low group in several databases while the reason remains unknown. In enrichment analysis, USP32 may be involved in the regulation of MAPK signaling pathway and Hippo signaling pathway which was worthy of being investigated deeply. Similar to the result of previous study, USP32 may also participate in the process of choline metabolism of cancer. Therefore, whether USP32 can regulate the metabolism processes of PDAC remains to be elucidated.

### 4.5. USP33

The roles of USP33 in tumor progression are complicated. USP33 is a classical mediator of Slit-Robo signaling pathway which is considered to be a tumor suppressor. In colorectal cancer, the USP33 expression was downregulated and related to tumor grade, lymph node metastasis, and prognosis. Moreover, USP33 could deubiquitinate and stabilize Robo1 and was responsible for the redistribution of Robo1 to the plasma membrane [42]. Additionally, USP33 was also discovered to regulate Slit-Robo signaling pathway and inhibit tumor migration in gastric cancer, breast cancer, and lung cancer [43–45]. On the contrary, Zhang et al. indicated that USP33 could stabilize HIF-2*α* to activate ERK1/2 and enhance cell stemness, tumor vascularization, and growth [46]. In retinoblastoma, USP33 was reported to regulate cell proliferation and inhibit apoptosis via stabilizing SP1 and activation of SP1/PI3K/Akt signaling pathway [47]. Our study revealed that the USP33 expression was higher in PDAC tissues than that in normal pancreatic tissues and also related to several clinicopathological features. However, just like USP32, USP33 also showed no significant correlation with prognosis of PDAC patients. Besides, USP33 expression level elevation was found in IPMA and IPMC without IPMN which indicated a value of precancerous lesions biomarker. Moreover, USP33 was also related to the cell stemness of PDAC cell which was similar to the result of the aforementioned study.

### 4.6. USP39

Several studies demonstrated the tumor-promoting role of USP39 in liver cancer. Initially, Pan et al. indicated USP39 silencing could cause proliferation inhibition and G2/M phase retardation [48]. Subsequently, USP39 was found to stabilize the SP1 expression to induce malignant biological behaviors of liver cancer [49]. Moreover, USP39 and TRIM26 balanced the expression of ZEB1 to regulate proliferation and metastasis of hepatocellular carcinoma [50]. USP39 was also involved in cell cycle of tumor cells. USP39 participated in regulation of G2/M phase and subsequent apoptosis of NSCLC cells through activation of p53 pathway [51]. In colorectal cancer, USP39 was reported to stabilize p21 to activate p21/CDC2/cyclin B1 pathway and promote their proliferation. Notably, USP39 influenced mRNA level rather than protein level of p21 in this study [52]. The similar mechanism of USP39 was also found in human glioma in which USP39 could induce ADAM9 mRNA maturation but not protein to promote glioma progression [53]. The present study indicated that the USP39 expression was higher in PDAC tissues than that in normal pancreatic tissues which was the same as the previous study [27]. Meanwhile, USP39 was also significantly associated with clinicopathological characteristics and survival of PDAC patients. GO enrichment analysis demonstrated that USP39 was robustly related to cellular processes which are related to cell cycle, and KEGG analysis further confirmed the potential value of USP39 in PDAC cell cycle. Therefore, the detailed mechanisms of USP39 in PDAC proliferation and cell cycle remain to be further elucidated.

Some limitations still existed in the present study. Firstly, the whole result of this study was based on bioinformatic analysis of online public databases which may be biased and required further verification of *in vivo* and *in vitro* basic experiments. Secondly, the PDAC data in different database were limited and different which caused the divergence and limitation of our results. Thirdly, the six-USPs were chosen just from bioinformatic analysis. Whether other USPs which have not been reported before possess effect on PDAC progression still needs in-depth study evidence. Finally, more online databases and tools should be used to accurately analyze the biological roles of USPs in PDAC.

## 5. Conclusion

In conclusion, this study comprehensively analyzed the expression, clinicopathological feature correlation, prognostic significance, promoter methylation, immune infiltration, genetic alteration and mutation, and enrichment pathways of six-USPs, namely, USP10, USP14, USP18, USP32, USP33, and USP39, in PDAC and identified the tumor-promoting role of six-USPs in PDAC using bioinformatic analysis. However, few studies have reported the roles and their potential mechanisms of six-USPs in PDAC initiation and progression. Therefore, a series of basic experiments *in vivo* and *in vitro* will be needed to further confirm the vital role of six-USPs in PDAC early diagnosis, prognostic prediction, and therapeutic target.

## Data Availability

The data used to support the findings of this study are included in the article.

## Supplementary Materials

Supplementary Materials Supplementary Figure 1: USP family members with no difference in expression between pancreatic ductal adenocarcinoma (*n* = 179) and normal pancreatic tissues (*n* = 171) in the GEPIA based on TCGA and GTEx databases. Supplementary Figure 2: transcriptional levels of six-USPs in 33 types of tumor and corresponding normal tissues. PAAD: pancreatic adenocarcinoma. Green words refer to low expression, and red words refer to high expression in tumor tissues. Supplementary Figure 3: methylation degree of the six-USPs in pancreatic ductal adenocarcinoma (PDAC) and normal pancreatic tissues and different pathological differentiation using the UALCAN database. (A) Methylation levels of the six-USPs in PDAC and normal tissues. (B) Methylation levels of the six-USPs in different differentiated degrees in PDAC. ^∗^*P* < 0.05, ^∗∗^*P* < 0.01; n.s.: not significant difference. Supplementary Figure 4: methylation degree of the six-USPs in different tumor staging and in pancreatic ductal adenocarcinoma (PDAC) patients with/without P53 mutation using the UALCAN database. (A) Methylation levels of the six-USPs in different tumor staging of PDAC patients. (B) Methylation levels of the six-USPs in PDAC patients with/without P53 mutation. ^∗^*P* < 0.05, ^∗∗^*P* < 0.01, ^∗∗∗^*P* < 0.001, and ^∗∗∗∗^*P* < 0.0001; n.s.: not significant difference. Supplementary Figure 5: the subgroup analysis of overall survival of the six-USPs in gender, mutation burden, and immune cell infiltration, including B cells, CD8+ T cells, macrophages, and regulatory T cells, in PDAC patients using the Kaplan-Meier plotter database. Supplementary Figure 6: the subgroup analysis of recurrence-free survival of the six-USPs in gender, mutation burden, and immune cell infiltration, including B cells, CD8+ T cells, macrophages, and regulatory T cells, in PDAC patients using the Kaplan-Meier plotter database.
