# Supplementary material for: USPs in Pancreatic Ductal Adenocarcinoma: A Comprehensive Bioinformatic Analysis of Expression, Prognostic Significance, and Immune Infiltration
Source: Biomed Res Int. 2022 Dec 20;2022:6109052. doi: 10.1155/2022/6109052 (PMC9794441; doi:10.1155/2022/6109052)
Supplement: Supplementary Materials — Supplementary Figure 1: USP family members with no difference in expression between pancreatic ductal adenocarcinoma (n = 179) and normal pancreatic tissues (n = 171) in the GEPIA based on TCGA and GTEx databases. Supplementary Figure 2: transcriptional levels of six-USPs in 33 types of tumor and corresponding normal tissues. PAAD: pancreatic adenocarcinoma. Green words refer to low expression, and red words refer to high expression in tumor tissues. Supplementary Figure 3: methylation degree of the six-USPs in pancreatic ductal adenocarcinoma (PDAC) and normal pancreatic tissues and different pathological differentiation using the UALCAN database. (A) Methylation levels of the six-USPs in PDAC and normal tissues. (B) Methylation levels of the six-USPs in different differentiated degrees in PDAC. ∗P < 0.05, ∗∗P < 0.01; n.s.: not significant difference. Supplementary Figure 4: methylation degree of the six-USPs in different tumor staging and in pancreatic ductal adenocarcinoma (PDAC) patients with/without P53 mutation using the UALCAN database. (A) Methylation levels of the six-USPs in different tumor staging of PDAC patients. (B) Methylation levels of the six-USPs in PDAC patients with/without P53 mutation. ∗P < 0.05, ∗∗P < 0.01, ∗∗∗P < 0.001, and ∗∗∗∗P < 0.0001; n.s.: not significant difference. Supplementary Figure 5: the subgroup analysis of overall survival of the six-USPs in gender, mutation burden, and immune cell infiltration, including B cells, CD8+ T cells, macrophages, and regulatory T cells, in PDAC patients using the Kaplan-Meier plotter database. Supplementary Figure 6: the subgroup analysis of recurrence-free survival of the six-USPs in gender, mutation burden, and immune cell infiltration, including B cells, CD8+ T cells, macrophages, and regulatory T cells, in PDAC patients using the Kaplan-Meier plotter database. [file 6109052.f1.docx]

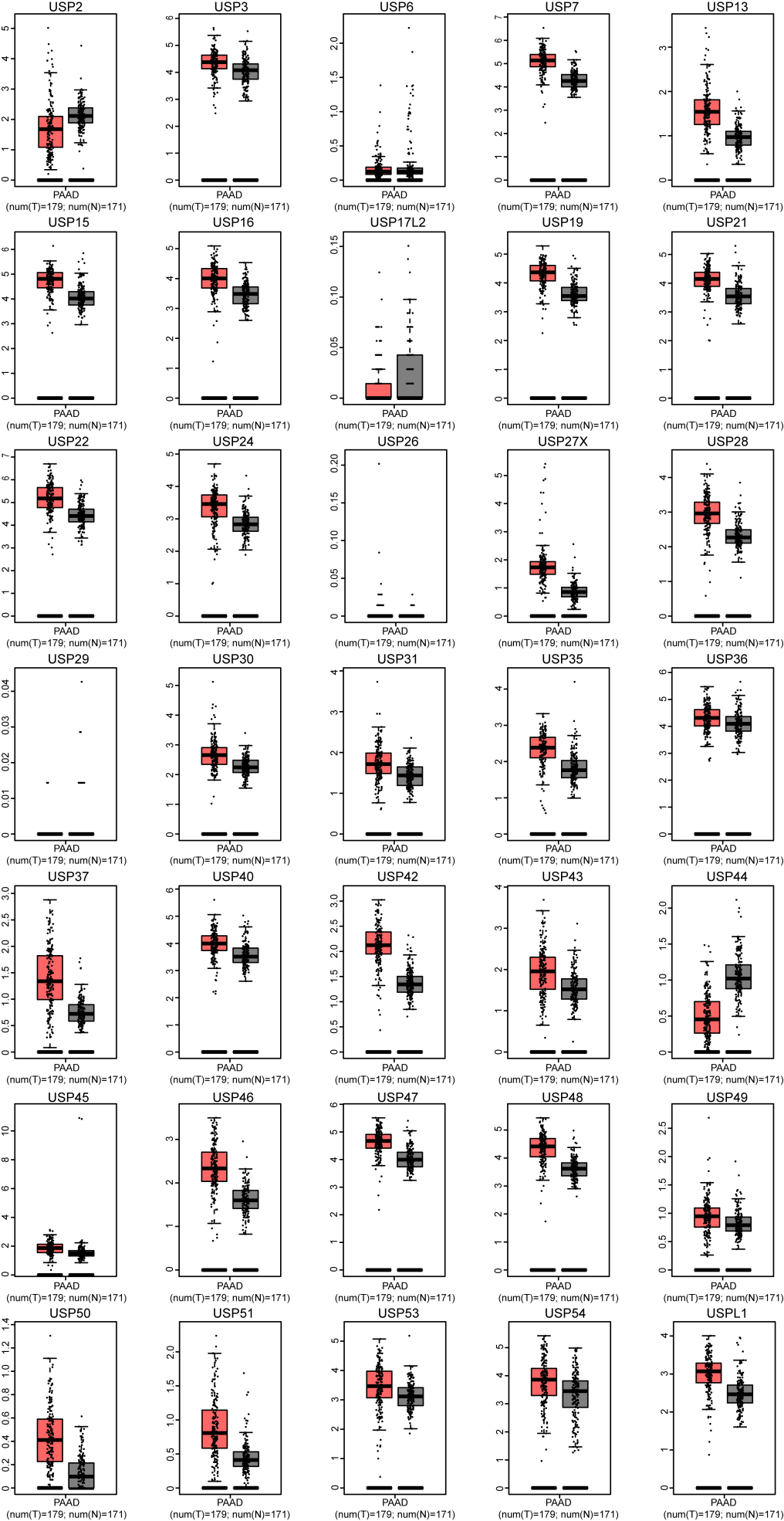
**Supplementary Figures**

**Supplementary Figure 1** USP family members with no difference in expression between pancreatic ductal adenocarcinoma (n=179) and normal pancreatic tissues (n=171) in the GEPIA based on TCGA and GTEx database.


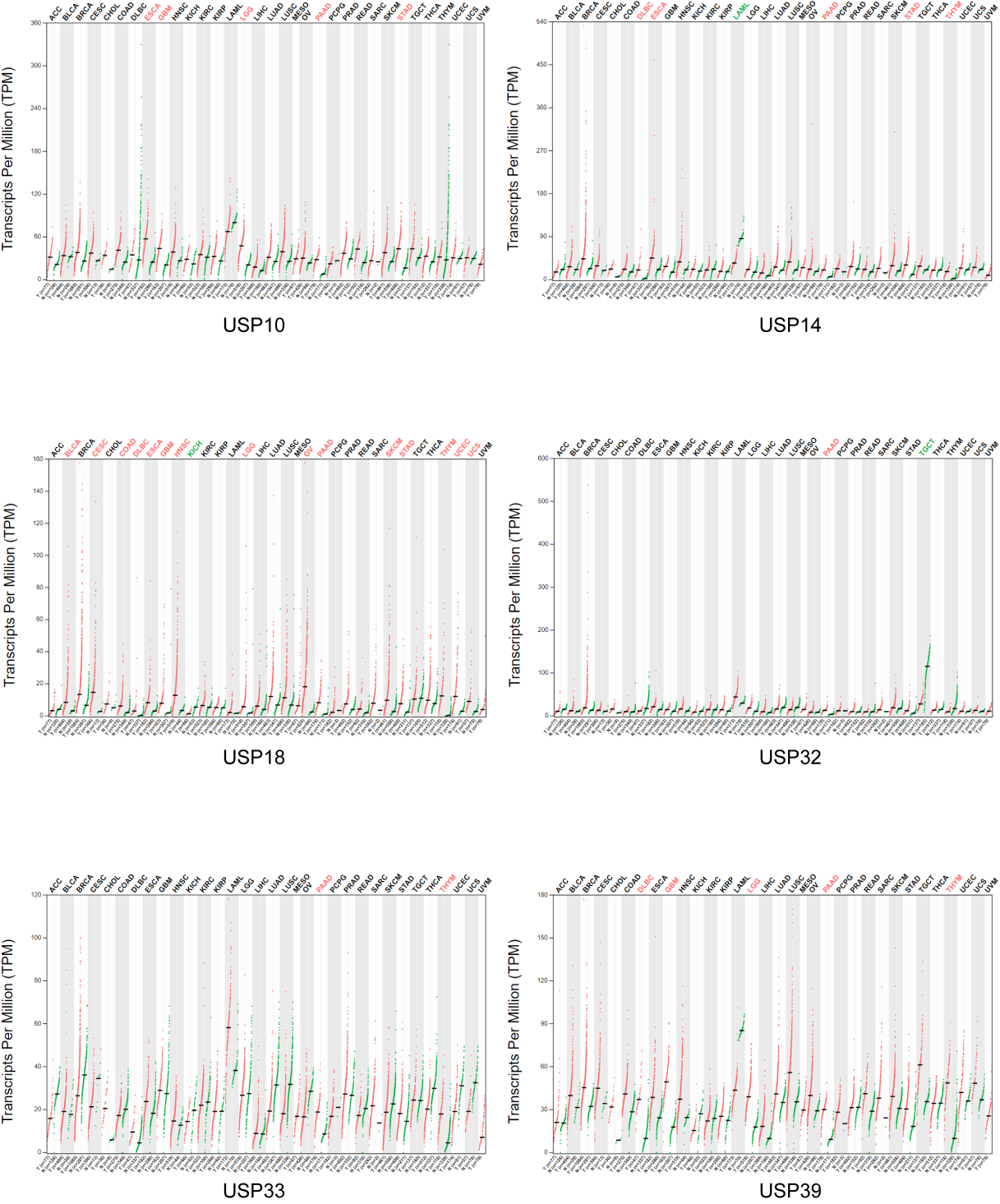
**Supplementary Figure 2** Transcriptional levels of six-USPs in 33 types of tumor and corresponding normal tissues. PAAD, pancreatic adenocarcinoma. Green words refer to low expression and red words refer to high expression in tumor tissues.


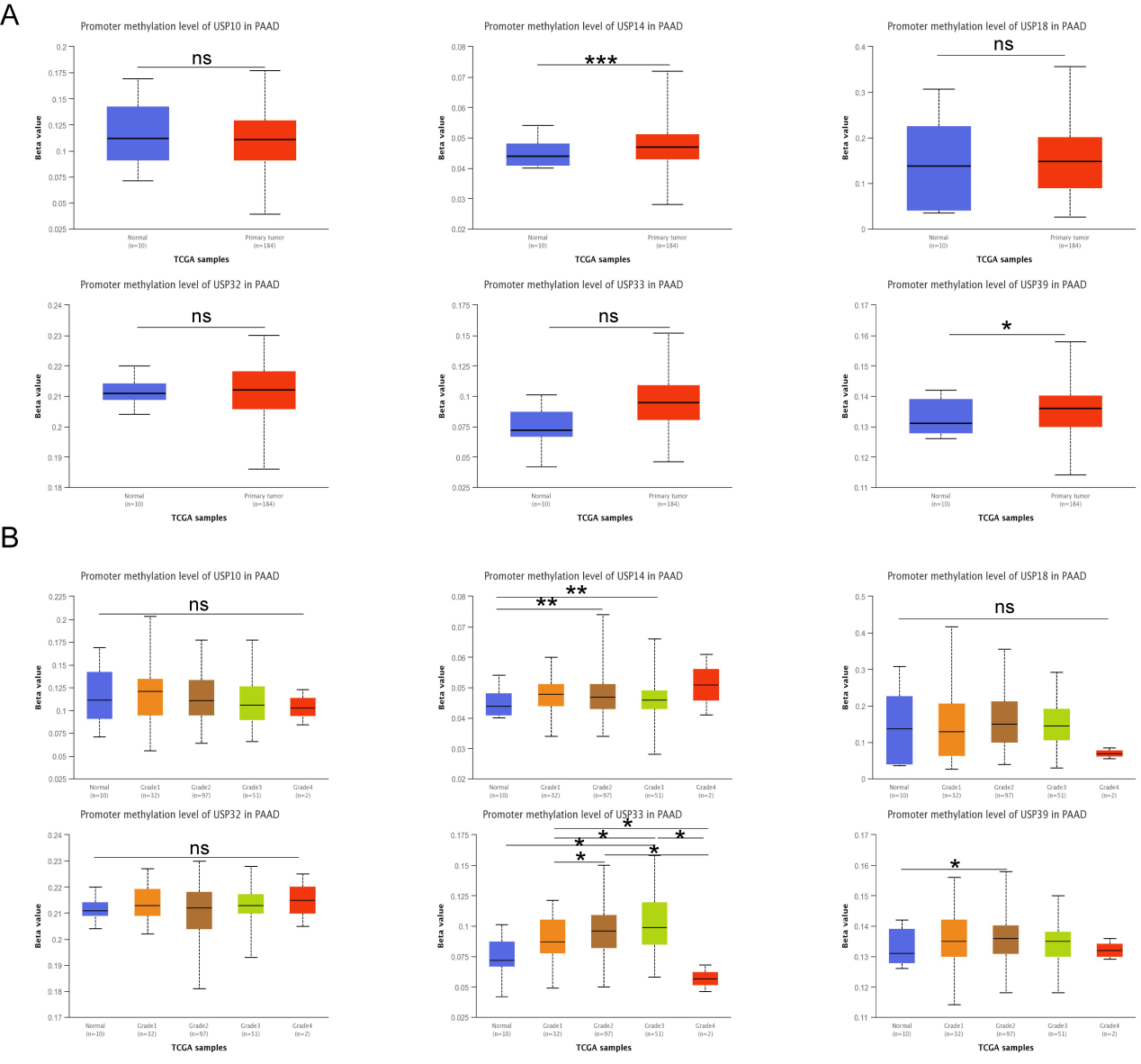
**Supplementary Figure 3** Methylation degree of the six-USPs in pancreatic ductal adenocarcinoma (PDAC) and normal pancreatic tissues, and different pathological differentiation using UALCAN database. (**A**) Methylation levels of the six-USPs in PDAC and normal tissues. (**B**) Methylation levels of the six-USPs in different differentiated degrees in PDAC. **P*<0.05, ***P*<0.01; n.s., not significant difference.


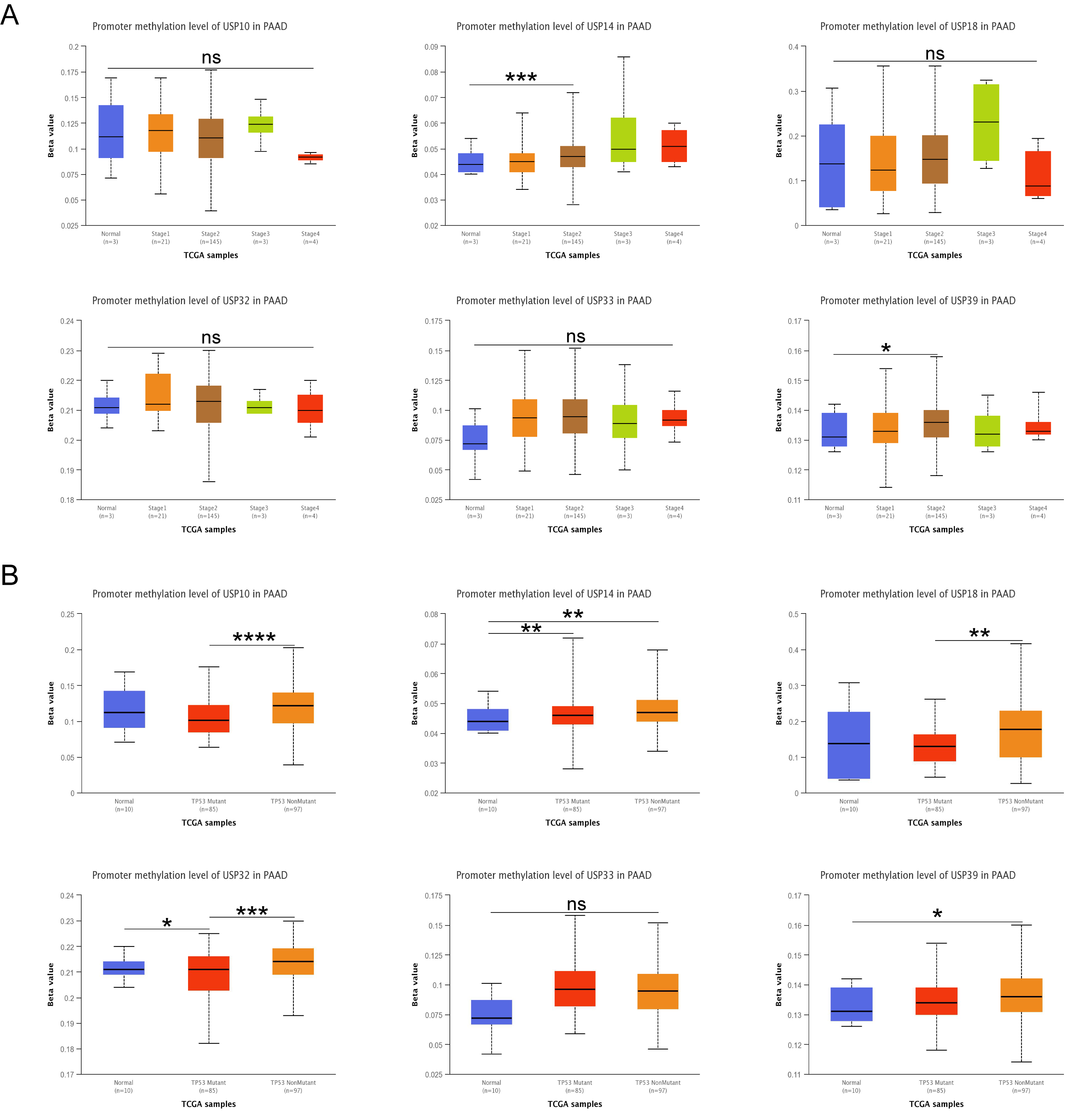
**Supplementary Figure 4** Methylation degree of the six-USPs in different tumor staging and in pancreatic ductal adenocarcinoma (PDAC) patients with/without P53 mutation using UALCAN database. (**A**) Methylation levels of the six-USPs in different tumor staging of PDAC patients. (**B**) Methylation levels of the six-USPs in PDAC patients with/without P53 mutation. **P*<0.05, ***P*<0.01, ****P*<0.001, ****P<0.0001; n.s., not significant difference.


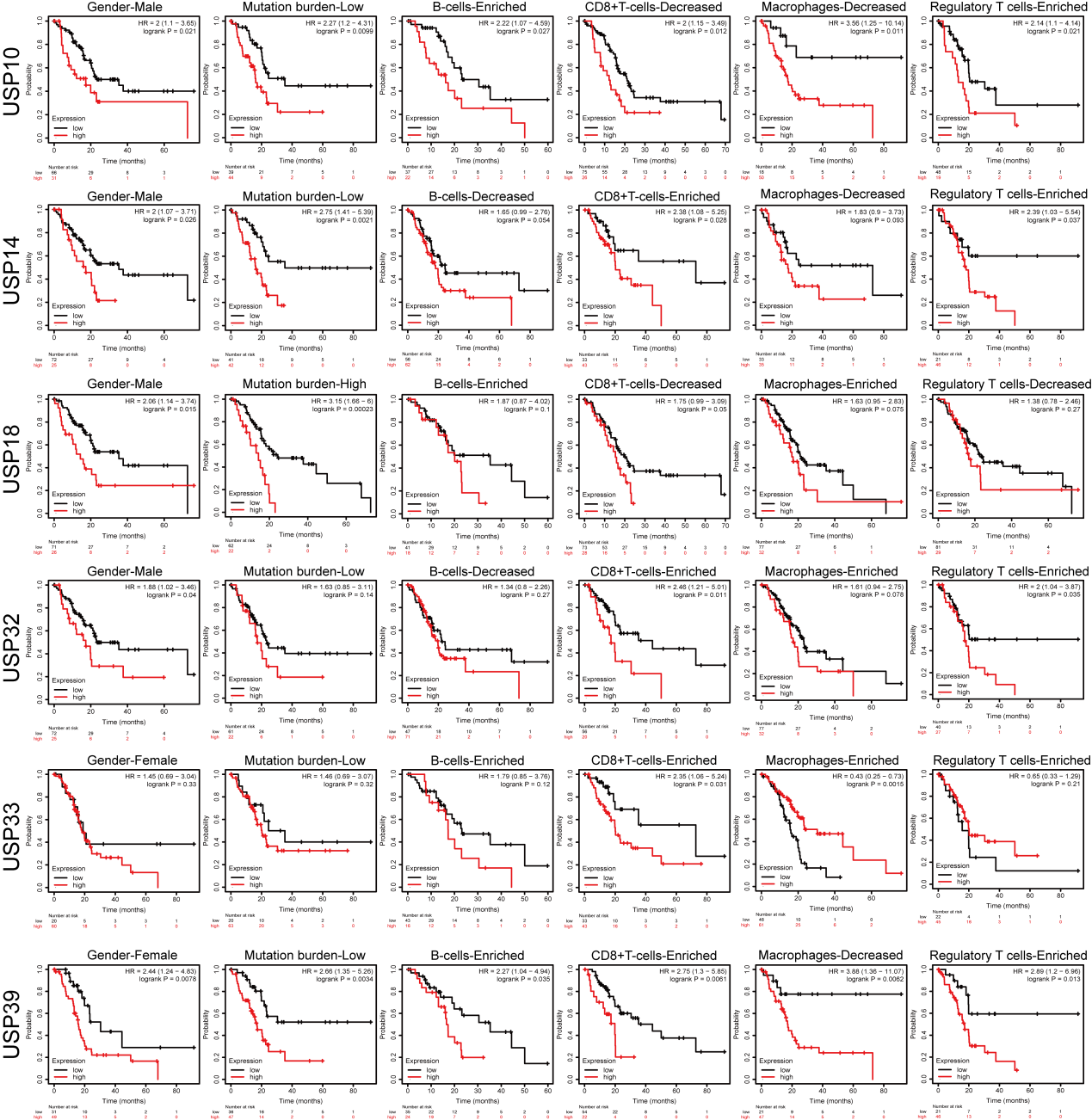


**Supplementary Figure 5** The subgroup analysis of overall survival of the six-USPs in gender, mutation burden, and immune cell infiltration, including B cells, CD8+T cells, macrophages, and regulatory T cells, in PDAC patients using the Kaplan Meier-plotter database.


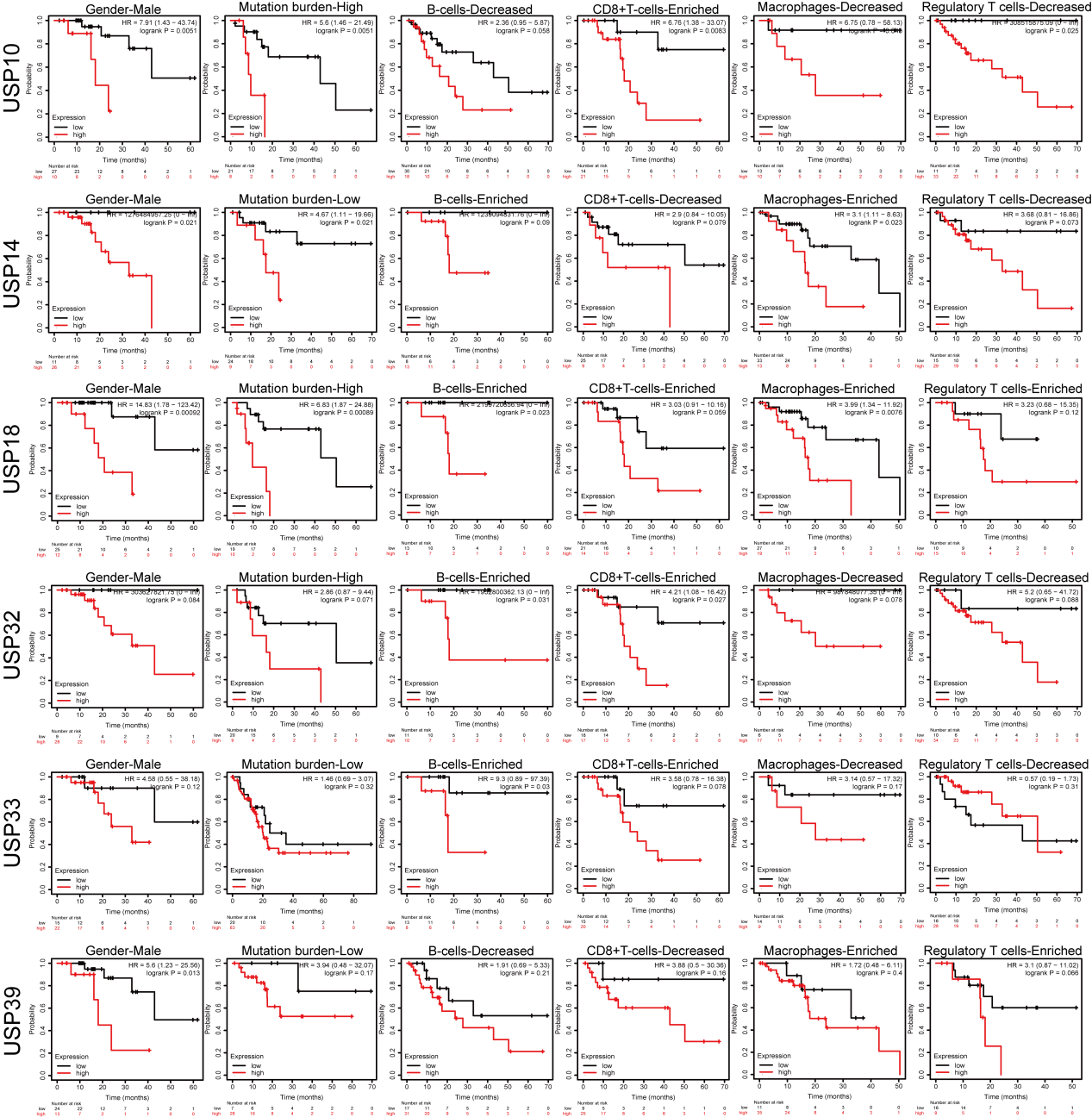


**Supplementary Figure 6** The subgroup analysis of recurrence-free survival of the six-USPs in gender, mutation burden, and immune cell infiltration, including B cells, CD8+T cells, macrophages, and regulatory T cells, in PDAC patients using the Kaplan Meier-plotter database.
